# Doctors’ experiences when treating doctor–patients: a scoping review

**DOI:** 10.3399/BJGPO.2023.0090

**Published:** 2023-11-29

**Authors:** Claire J Hutton, Margaret Kay, Penny Round, Chris Barton

**Affiliations:** 1 Department of General Practice, School of Public Health and Preventive Medicine, Monash University, Melbourne, Australia; 2 General Practice Clinical Unit, Faculty of Medicine, The University of Queensland, Brisbane, Australia; 3 School of Curriculum Teaching and Inclusive Education, Faculty of Education, Monash University, Melbourne, Australia

**Keywords:** general practitioners, physicians, family, physician’s role, physician–patient relations, physicians, practice patterns

## Abstract

**Background:**

To work effectively, doctors need to look after themselves. They often delay seeking medical care for a range of reasons. Once they do, there is evidence that the doctors treating them (’treating doctors’) can struggle to provide optimal care.

**Aim:**

To examine existing literature on what is currently known about experiences for treating doctors, in particular GPs, when their patient is also a doctor.

**Design & setting:**

A scoping review of articles written in English.

**Method:**

Using the JBI methodological framework for scoping reviews, five databases (MEDLINE, PsycINFO, CINAHL [Cumulative Index to Nursing & Allied Health], Google Scholar, and Scopus) were searched from the database start date until 31 December 2022. Qualitative and quantitative studies reporting the treating doctor’s experience, guidelines for treating doctors, expert opinion articles, and editorials were included. Grey literature was considered, searching the first 10 pages of two Google searches.

**Results:**

Forty-eight articles from eight countries met inclusion criteria, of which 12 were research studies. The main areas of focus were as follows: affective responses, which included anxiety about being criticised, concern about upsetting the doctor–patient, and discomfort regarding the acknowledgement that doctors get sick; relational factors, which included boundary issues, over-identifying with the doctor–patient, treating them as a colleague rather than a patient, and role ambiguity; confidentiality, which incorporated both affective and relational aspects; and influence of medical culture and socialisation on dynamics between treating doctor and doctor–patient. These findings have been distilled into a list of key suggestions for the treating doctor.

**Conclusion:**

Doctors can find treating doctor–patients anxiety-provoking and challenging. The sources of this discomfort are multifaceted, and more empirical research is needed to better understand and address the complex relationship between treating doctor and doctor–patient.

## How this fits in

This review identified a wide range of articles (albeit limited research studies) exploring doctors’ experiences when treating doctor–patients. Treating doctors experienced anxiety, boundary issues, and role ambiguity when caring for doctor–patients. This review has summarised the current guidance for treating doctor–patients within the literature and has highlighted the paucity of research in this area and the limited accessibility of this guidance. More research is needed to better understand how doctors (especially those in general practice, the usual healthcare system entry point) can improve their care of doctor–patients.

## Introduction

GPs have a dual role in providing preventive and primary health care, as well as being the entry or referral point to secondary medical care. Building a strong relationship with a GP during visits for ‘minor’ ailments and preventive care is important in itself, but becomes much more so when serious health issues arise. Doctors often do not have an independent GP, despite strong recommendations by medical boards that they do.^1–3^ Those who do have a GP tend to see them infrequently, for a variety of reasons, including the ability to self-treat and to access corridor consultations with colleagues. A systematic review on doctors’ health access and the barriers they experience categorised barriers into those related to the profession, the patient, and the provider.^1^ Professional barriers included the work environment, with its structural demands and lack of time, the ability to self-treat, and the culture of medicine, which trains doctors through a process of professional socialisation that produces an ethos of invulnerability.^4^ Patient barriers included embarrassment around being a patient, which may contribute to doctors avoiding, denying, or rejecting the role of patient.^5–8^ GPs themselves can struggle to seek timely health care. A 2019 survey by the Royal Australian College of General Practitioners found that 41% of GPs had delayed seeking treatment or care in the past 2 years, and more than one-quarter of those (*n* = 135, 28%) stated that this was owing to feeling uncomfortable seeking care from other GPs (although reasons for this discomfort were not explored).^9^ Provider barriers (those predominantly under the control of the medical care provider) included the discomfort that doctors can experience when delivering health care to a colleague.^10^ Kay^11^ reported a common view that many, if not most, doctors are ambivalent about accepting another doctor as a patient, but data are lacking.

This scoping review focused on the doctor–patient relationship, when the patient is a doctor, with an emphasis on the perspectives and experiences of the treating doctor. Preliminary literature searches, using Google Scholar and PubMed, revealed a predominance of ‘expert opinion’ articles, with few research studies. Peters *et al* ^12^ noted that scoping reviews are especially useful when the literature is multifaceted and heterogenous. A preliminary search of JBI Evidence Synthesis, Epistemonikos, PROSPERO, and Cochrane Database of Systematic Reviews (January 2021) found no relevant systematic or scoping reviews on this topic.

The aim of this review was to map the existing evidence and provide an overview of the experiences and challenges for the treating doctor when caring for a doctor–patient for any health issue, either physical or mental. The concepts used by researchers to describe and explain their findings were also explored. Gaps in empirical evidence were identified to inform further research, with the broader goal of helping doctors to become more comfortable and effective in caring for medical colleagues.

## Method

The review followed the JBI methodology for scoping reviews,^12^ using the Preferred Reporting Items for Systematic Reviews and Meta-Analyses (PRISMA) extension for scoping reviews checklist. The initial search focused on the provision of primary health care by GPs to doctor–patients, but few articles were found. The search was broadened to all treating doctors, when their patient is another doctor.

### Data sources and search strategies

Medical and health literature databases (MEDLINE and PsycINFO both via the Ovid platform, CINAHL by EBSCO host, Google Scholar, and Scopus) were searched, from their start date until 31 December 2022 (see Supplementary Figure S1 for MEDLINE search strategy). Subsequent searching of other databases was adapted from this strategy. Additional articles were identified from the reference lists of studies selected for full-text review.

Qualitative, quantitative, and mixed-method formats describing or measuring aspects of the treating doctor or doctor–patient experience were included. Guidance for treating doctor–patients, expert opinion articles, editorials, and letters to the editor were also considered (see Box 1 for inclusion criteria). The first 10 pages of two Google searches (’doctors[physicians] treating other doctors[physicians]’ and ‘treating doctor[physician]–patients’) were screened to identify additional relevant peer reviewed or grey literature. Only articles in English were included.

Box 1 **Inclusion criteria**

- Treating clinician is a medical practitioner
- Patient is a doctor or medical student
- Studies (quantitative, qualitative, mixed methods, case studies, systematic reviews) and articles (editorials, letters, expert opinion articles) that describe, explore and/or measure aspects of the doctor’s experience when their patient is also a doctor
- Published in English

### Study selection, data extraction, and synthesis

The lead author (CH) undertook the database search and identified articles to be collated and uploaded into the web-based management software Covidence. Two reviewers (CH and CB) independently screened titles and abstracts for assessment against the inclusion criteria. Potentially relevant articles were retrieved in full and assessed in detail against the inclusion criteria by CH, PR, and SV.

The articles were extracted by SV and CH using a custom data-extraction table. An iterative process was used, which was updated as articles revealed useful categories. CB reviewed the extracted data from the first 20% of articles to check consistency and quality. Given the heterogeneity of articles that met inclusion criteria, narrative synthesis was used to summarise findings.

## Results

**Figure 1.**
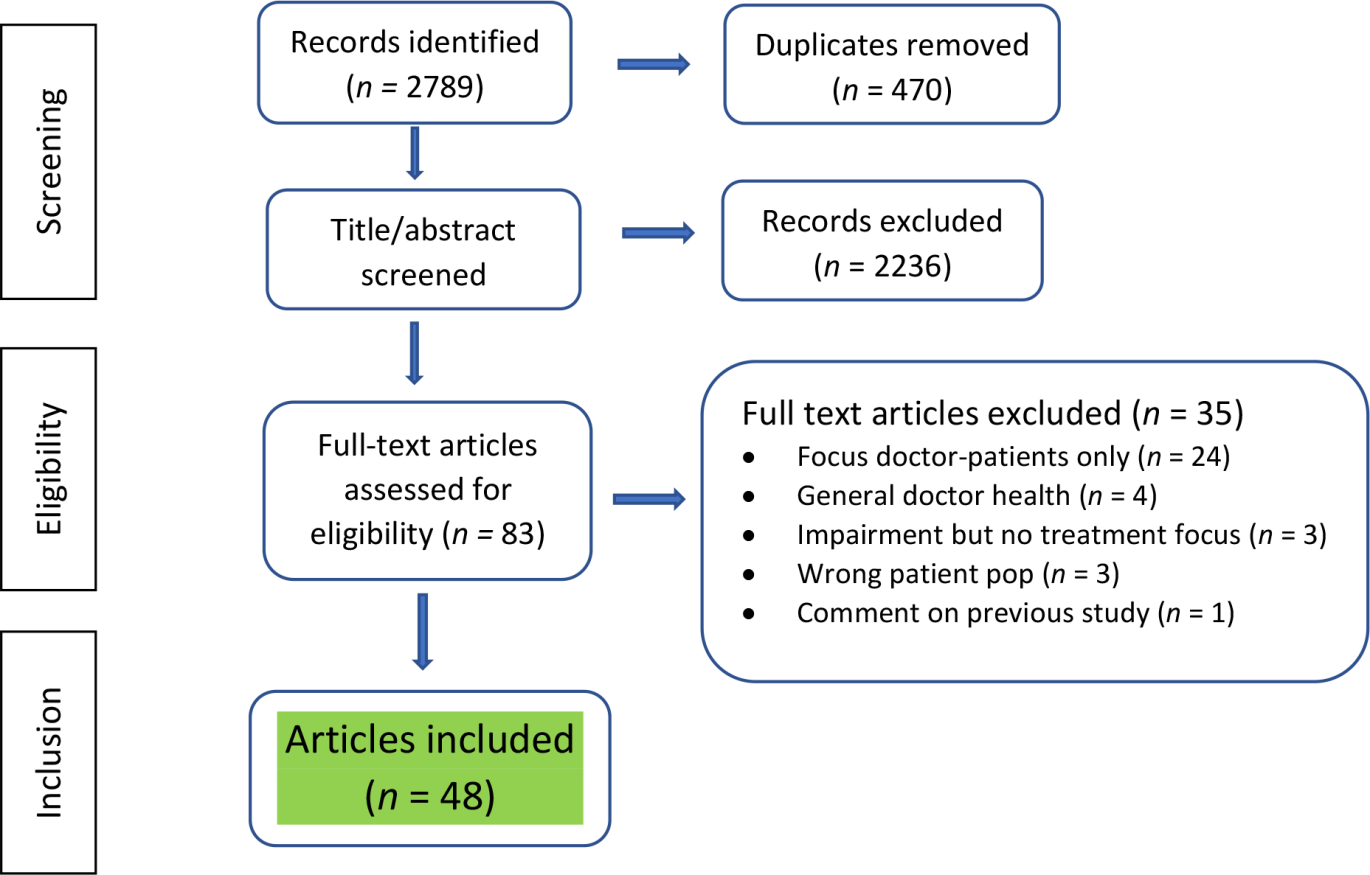
PRISMA flowchart for study selection process. Pop = population.

A total of 2319 title and abstracts and 88 full-text articles were screened (see Figure 1). Data were extracted from 48 articles from eight countries, including 12 research studies (see Table 1). Six of these studies primarily addressed the experiences of the treating doctor and three included both the doctor–patient and treating-doctor perspective.

**Table 1.** Study type

Study type	Number	Article reference numbers
Book chapter	2	^5,23^
Empirical, qualitative	11	^4,7,13–17,19–22^
Empirical, quantitative	1	^18^
Expert opinion	20	^10,24,26–28,32,33,35–38,40,45–47,49–51,57,58^
Perspective (based on case studies or vignettes)	3	^6,8,44^
Editorial or commentary	4	^29,30,39^^,^^43^
Letter	3	^31,48,59^
Literature review	4	^25,34,41,42^

Of the 12 empirical studies, five focused on primary care or general practice, four recruited doctors from a range of specialties (two of these included primary care), one focused on palliative care, one on oncology, and one on psychiatry (see Table 2). In some countries, specialists may be the main care provider and it was determined that the breadth of experience of caring for doctors was important to capture. All studies except one used qualitative methodology (interviews or focus groups).

**Table 2.** Profile of studies: country of authors, year of publication, discipline setting or specialty

Attribute	Non-research articles(*n* = 36)	Article reference numbers	Empirical studies(*n* = 12)	Article reference numbers
Country				
US	14	^6,8,25,28,31–34,37,39–41,45,49^	6	^15–19,22^
UK	8	^23,27,29,35,42,48,51,57^	3	^7,14,21^
Australia	7	^26,30,36,46,47,50,58^	1	^13^
New Zealand	2	^5,43^	2	^4,20^
Canada	2	^10,59^		
Other (Belgium, Israel, Japan)	3	^24,38,44^		
Year of publication				
1963–1980	1	^37^	2	^17,22^
1981–1990	5	^23,24,28,31,32^		
1991–2000	7	^5,10,40,45,46,51,57^	1	^7^
2001–2010	13	^8,26,27,34,35,38,39,41,42,47,50,58,59^	5	^4,13,14,19,21^
2011–2022	10	^6,25,29,30,33,36,43,44,48,49^	4	^15,16,18,20^
Setting or specialty				
General practice or primary care	6	^5,31,35,47,51,57^	5	^4,13–15,18^
General	22	^6,10,23,25–29,32,34,36–38,40,41,43,45,46,48,50,58,59^	4	^7,17,19,22^
Hospital or cancer centre	2	^39,44^	1	^16^
Mental health or psychiatry or addiction	5	^24,30,33,42,49^	1	^20^
Palliative care	1	^8^	1	^21^

Studies were diverse in their focus, including the following:

- Experiences of doctor–patients,^4,13^ including comparing these to ethical guidelines;^14^
- Differences, challenges, and strategies when treating doctor–patients;^15–18^
- Challenges in the relationships between the treating doctor and doctor–patient. One study focused primarily on doctor–patients,^19^ another on the psychiatrist–patient relationship,^20^ a third on the palliative care setting;^21^
- Medical socialisation and culture: examining the extent to which social rather than medical factors impact on a doctor’s decision regarding choosing a treating doctor,^22^ and exploring the impact of the notion that doctors should not get sick.^7^

While the empirical studies added strength to the findings, the following discussion incorporates the issues explored in all 48 articles.

### Themes identified

The following three overarching themes were idenified:

1. Affective responses of the treating doctor;
2. Relational factors, including boundary issues and role ambiguity;
  - A sub-theme, confidentiality, encompassed elements of both 1 and 2;
3. The influence of medical culture and socialisation.

### Affective responses

While doctors may feel flattered to be chosen by a colleague to provide care,^16,17,23–25^ they can also find it awkward^10,16,26^ and intimidating, especially providing care to a doctor–patient whose knowledge or experience is similar or greater than their own.^10,27–30^ They can feel less able to form independent judgements about the best treatment^8,31^ and may defer to what they perceive as the doctor–patient’s superior knowledge.^32,33^

Treating doctors can feel anxious about the possible scrutiny of their performance^16,17,26,28,34^ and fear making an error or missing something important,^26,33,35^ and the criticism that may ensue from the wider medical community.^20,30,32,36^ These concerns can lead to over-investigation, ordering more tests to demonstrate their competence and reduce likelihood of errors.^5,32,37,38^ First noted by Rhodes almost 40 years ago, this remains a concern in more recent literature.^23^ The only quantitative study^18^ found 42% of primary care doctors would order more tests and procedures, if their patient was another doctor, while a qualitative study^16^ of oncology specialists found none reported differences in testing for their doctor–patients.

Another source of anxiety was fear of upsetting or embarrassing the doctor–patient. This fear can result in a deviation from standard evaluation approaches, by omitting questions about relevant risk factors and personal habits,^5,6,8,25,28,29,32,36,38,39^ such as alcohol and other drug use, relationships, sexual problems, mental health and self-treatment, or by under-testing, especially avoiding intrusive and awkward tests such as rectal exams.^8,29,38,40,41^

The treating doctor may also hesitate to ask questions about mental health or substance use, out of concern that the doctor–patient’s answers might indicate a risk of harm, to themselves or their patients, which may then require reporting to regulatory authorities.^26^ If this step is taken, there can also be worry about *‘victimising the doctor if there is insufficient evidence*’.^42^

Doctor–patients (like all patients) can use ‘selective disclosure’^30^ when giving a medical history, either owing to an a priori self-diagnosis, or an attempt (consciously or unconsciously) to avoid an unwanted diagnosis.^10,30,31^ Unlike with other patients, treating doctors may fail to interrogate that history in the usual way, assuming the doctor–patient will share all relevant information.^10,30,32,43^

Doctor–patients may have difficulty relinquishing authority and control.^8,21,22,30,39,41,44^ If the treating doctor is already experiencing anxiety and fear of scrutiny, this makes it easier to allow their patient to make treatment decisions, perhaps abdicating their responsibility, while potentially resulting in less than optimal care.

Treating doctors can also experience frustration with their doctor–patients, if they felt they were being manipulated into a treatment plan against their better judgement.^16,31,33^ As it can be time-consuming to treat a doctor–patient, the increased workload can also result in feelings of irritation.^26,27,32^

### Relational factors

Two sub-themes were identified, boundaries and role ambiguity.

#### Boundaries or over-identifying with the doctor–patient (or fear of doing so)

While some treating doctors felt that over-identifying with doctor–patients can improve rapport and empathy,^20,25^ concern about over-identification can result in doctors distancing themselves from their doctor–patient to maintain objectivity.^8,24^ One article suggested distancing happens because the patient represents a mirror reflection of the treating doctor’s own unconscious fears about illness.^28^ Another saw it as self-protection from suffering, should the patient deteriorate.^8^ Walking the fine line between over-involvement and objective detachment is challenging.^34^ Some treating doctors reported that they provided their doctor–patient a greater degree of accessibility, including giving their personal phone number.^16,21^

The myth of physician invulnerability to illness appeared regularly in the literature.^20,32,34,45,46^ Seeing a doctor–patient forced the treating doctor to acknowledge that doctors do get sick, and perhaps to confront one’s own mortality. Concern was expressed that if the treating doctor failed to recognise such beliefs, this could lead to anger at the peer’s ‘weakness’,^24^ minimisation of testing,^31^ high expectations of compliance and recovery,^7^ or collusion with the patient in denying the possibility of serious illness.^24,32^

#### Role ambiguity

Role ambiguity was a strong sub-theme emerging from empirical studies. Shared understanding of medical terminology could be used by the doctor–patient to exercise greater control in treatment decisions, and mislead the treating doctor into believing their patient has a greater understanding than is the case.^14,15,29^ Otte *et al* ^38^ called this ‘terminology-itis’. It can result in the treating doctor providing inadequate information (regarding diagnosis, treatment, or prognosis), by assuming the patient has sufficient knowledge^6,8,23,34,37,39,47^ or being worried about insulting them.^5,28,32^ The doctor–patient may be too embarrassed to question if they don’t understand the medical terms,^5,8,23,48^ which can lead to uninformed consent for procedures,^5^ and weakening of the therapeutic relationship.^8,34^ Role ambiguity can also result in the treating doctor mistakenly accepting the doctor–patient’s opinion about diagnosis or treatment.^5,25^

Both boundary confusion and role ambiguity can result in the treating doctor providing limited emotional support, ignoring distress, or assuming the doctor–patient does not need such support.^7,19,28,32,42,45^

### Confidentiality

A distinct sub-theme of confidentiality was relevant to both the anxiety experienced by doctors, and the boundary issues.

Discussing cases with colleagues to get another opinion is a widely accepted practice, but can be a concern for some treating doctors.^10,33^ While they may be in greater need of advice and reassurance,^32^ owing to the anxiety and fear of scrutiny discussed above, they are also aware that their doctor–patient may be known to colleagues. Avoiding such consultation^37^ could impact the doctor–patient’s care.

Treating doctors can find themselves under pressure to provide confidential information to other colleagues uninvolved in the doctor–patient’s care.^21^ The ‘VIP’ status of doctor–patients may also result in a treating doctor being tempted to boast to colleagues about their patient, seeing it as a reflection of their competence.^23^

### Influence of medical culture and socialisation

Many authors conceptualised their findings from the perspective of the influence of medical culture and socialisation. Constructs included invulnerability and the denial of illness,^47^ the desire to underestimate the severity of illness in the doctor–patient,^49^ helping the doctor–patient to deny or postpone treatment,^24^ or struggling to probe health beliefs, which may lead doctor–patients to over or underdiagnose their own illness.^27^

Treating doctors may not wish to comment on their doctor–patient’s self-treatment.^19^ Doctor–patients may be reluctant to share their differing opinion and avoid challenging their treating doctor. Stanton and Randal’s^20^ study reported that many doctor–patients attributed this to their desire to be a ‘good patient’, while treating doctors were not aware of this desire.

Bynder explored how doctors choose their treating doctor.^22^ Becoming a patient means being in a subordinate position. This may be more tolerable if the chosen treating doctor has equal (or even lower) status than the doctor–patient. The authors described status as correlating with competence, and concluded that their responders were assessing *‘loss of quality of care to be less costly than the loss of social rank’*.^22^

### Guidance for working with doctor–patients

Eighteen articles, all except one^15^ based on expert opinion, included advice on how best to care for doctor–patients (Box 2). Three highlighted how the doctor–patient should be treated differently to regular patients; contacting them before the appointment to discuss how long they might need, where to wait, and confidentiality,^50^ and being flexible and understanding of work demands by prioritising and expediting the consultation^30,51^ because doctor–patients often delay seeking help. However, most suggestions focused on how a doctor–patient should be treated the same as other patients.

Box 2 **Commonly noted suggestions for consultations with a doctor–patient**

- Avoid corridor consultations.^10,28,35,57^
- Allow adequate time to get to know your patient and to discuss their issues, listening without interrupting.^10,39,45,47,58^
- Ask the ‘difficult’ questions around substance use, sexual and mental health, suicide risk, and self-medication.^10,35,43,49,51,57,58^
- Include the usual testing routine.^10,30,41,45,47,51,57,58^
- Have a heightened recognition of ethical concerns (confidentiality, medical records) and discuss early.^39,41,45,48,50,57,58^
- Discuss the treatment plan in detail,^45,47^ and follow-up as per usual practice.^10,58^
- Acknowledge and allow for individual differences in how doctor–patients and treating doctors negotiate their relationship and the degree to which treating doctors take charge, or involve their patient in decision making.^15,43,45,47,59^
- If overly-anxious, don’t accept doctor–patients.^29,30,41,45^

## Discussion

### Summary

This review identified that the existing knowledge about the experiences of treating doctors caring for a doctor–patient is for the most part founded on expert opinion. While a wide variety of articles were found, discussing the subject from a range of perspectives, few were empirical studies. The majority of the empirical articles included data from GPs.

Doctors, including GPs, often find the experience of treating another doctor challenging. Concern about making errors, being judged, and not wanting to embarrass a peer can result in limited history-taking, and under or overtreatment. This review has provided important insights that will support treating doctors in the delivery of quality care to their doctor–patients.

A major issue cited in the literature is the treating doctor’s assumption that a doctor–patient needs less explanation about their illness and treatment,^52^ which can result in the patient experiencing isolation and receiving limited information or reassurance. Treating a colleague involves complex socio-cultural dynamics with the risk of collusion, challenges to the sense of invulnerability to illness, and difficulties in delivering empathic care.

Maintaining appropriate boundaries while delivering empathic care required careful balance. Some treating doctors stated they were more accessible to their doctor–patients by, for example, providing their personal phone number; however, these issues were not explored in depth. It is likely that doctors working in more regional and rural areas would find these boundary issues even more complex to navigate, as they regularly cross paths as colleagues while maintaining a distinct therapeutic relationship.

### Strengths and limitations

To the authors’ knowledge, this is the first scoping review focusing specifically on the delivery of health care of doctors from the treating-doctor perspective. Given the limited articles, articles that described treating-doctor issues were included even when their focus was on the doctor–patient.

Two search term obstacles were noted. First, most results from the term ‘doctor–patient’ refer to the interaction, relationship, and communication between a doctor and their patient (rather than a doctor who is a patient). Second, the terms ‘doctor’s doctor’ and ‘physician health’ are frequently used to refer to mental health or impairment, rather than health more generally.

The decision to include expert opinion as well as research could be seen as a limitation. McArthur *et al* ^53^ noted expert opinion can either complement empirical evidence, or where there is none, exist as the best available evidence. Overall, there was considerable consensus between the expert opinion and research studies in this review.

This review was limited by only including publications in English. While recognising that there are cultural and linguistic differences that would influence the therapeutic relationship between treating doctors and their doctor–patients who work in other countries, it is very likely that the medical professional culture is likely to be dominant when considering this therapeutic relationship. The findings of this review are likely to resonate with medical practitioners in other non-English speaking countries, although this would require further research to confirm.

### Comparison with existing literature

There has been limited theoretical exploration to help understand the impact on the doctor–patient relationship when the patient is also a doctor. Bynder^22^ utilised social exchange theory^54^ to explore how social rather than medical factors influence who a doctor chooses to be their doctor. Interpersonal processes have also been examined through a broader socio-cultural lens, looking at the challenges for both treating doctors and doctor–patients, and the impact of medical culture and professional identity.^4,8,13,14,19^ Some studies focused on intrapersonal factors common to doctors, such as obsessionism and perfectionism.^8,19,30^ This heterogeneity of approaches, and the majority of articles written solely from an ‘expert opinion’ position, has highlighted a need for further research, in particular exploring *‘the interconnections between identity, work, and health*’^19^ within the medical profession.

### Implications for practice

This scoping review has demonstrated that doctors who treat other doctors can struggle to provide optimal care. There is a clear need for further empirical and theoretically driven research specifically focusing on the doctor treating other doctors. Much expert opinion is written with the psychiatric lens, rather than the lens of primary care. Developing a stronger evidence base for the challenges for GPs when treating doctor–patients is important. General practice is usually the first entry point into the healthcare system. Many professional organisations, colleges, and regulatory bodies recommend that doctors have their own GP.^2,3^ If a GP is able to show equanimity and empathy to their colleague, while still responding as a doctor, this is likely to support the doctor–patient’s willingness to engage effectively in this therapeutic relationship.

Some of the guidance for treating doctor–patients is difficult to access. The barriers to adhering to best practice are rarely addressed. Lam^6^ noted that the medical curriculum should include training about the complex counter-transference that occurs between treating doctors and doctor–patients. Evidence-based training for doctors (especially GPs) who treat other doctors as patients is crucial to improve the health care they provide.^55,56^

## Data

The dataset relied on in this article is available from the corresponding author on reasonable request.
